# T1 bladder cancer on restaging transurethral resection should be treated with immediate cystectomy

**Published:** 2007

**Authors:** Gagan Gautam, Rajeev Kumar*

**Affiliations:** Department of Urology and Renal transplant, Fortis Flt. Lt. Rajan Dhall Hospital, Vasant Kunj, New Delhi, India. E-mail: rajeev02@gmail.com

## SUMMARY

In order to evaluate the impact of pathological findings at restaging transurethral resection (TUR) on tumor recurrence and stage progression, a cohort of 352 patients with T1 bladder cancer underwent a second or restaging TUR after a two to four-week interval. All patients received induction bacillus Calmette-Guerin (BCG) therapy and 88% were followed for five years or more. Maintenance regime for BCG was not instituted in any of the patients. The median (range) follow-up was 7.5 (1-14) years. Pathological findings on restaging TUR were correlated with tumor features, recurrence rates, stage progression frequency and progression-free survival. Of the 352 patients with T1 tumors 203 (58%) had residual tumor on restaging TUR, including 92 (26%) with residual non muscle-invasive (T1) cancer. During the first five years, 66% of cases recurred and 35% progressed in stage. Recurrence and progression rates were 48% and 8% for patients with no residual tumor on restaging TUR, 69% and 37% for those with carcinoma *in situ* (CIS), 79% and 16% in cases with residual TaG3 tumor and 88% and 82% in patients with residual T1 tumor. Therefore, of the 92 patients with residual T1 cancer on restaging TUR, 75 (82%) progressed to muscle invasion within five years compared to 49 of 260 (19%) who had no or non T1 tumor detected on restaging transurethral resection. In a multivariate analysis of risk factors, restaging TUR pathology was the most important single determinant associated with tumor progression (Hazard ratio, HR - 6.7, *P*-0.000) followed by the presence of complete response to BCG at first cystoscopy performed three to six months after TUR (HR-2.7, *P*-0.001), presence of multiple tumors (HR-3.3, *P*-0.04) and high-grade tumors (HR-3.1, *P*-0.05). The authors concluded that restaging transurethral resection identifies patients with T1 bladder cancer who are at high risk for early tumor progression and that this finding justifies an immediate cystectomy in this group of patients to prevent an adverse outcome in the long term.

## COMMENTS

Tumors invading the lamina propria (T1) constitute about one-third of all superficial bladder tumors and have recurrence and stage progression rates (with bladder conservation) of 70% and 30-50% respectively within 5-10 years of initial presentation.[1] Some of the factors that portend a poorer prognosis in terms of stage progression rates are multiple tumors, Grade 3 morphology, large size (more than 3 cm), CIS and tumor at first follow-up cystoscopy.[2]

A restaging TUR for patients presenting with T1 tumors detects residual disease in a significant proportion of patients[3] and improves the initial response to BCG, thereby potentially delaying stage progression.[4] Although some authorities now recommend an aggressive management of T1 G3 tumors with radical cystectomy, most of the T1 tumors are still treated with BCG and bladder preservation. Radical cystectomy is usually performed only when muscle invasion is documented in subsequent biopsies or if the tumor/coexisting CIS is found to be refractory to BCG. This study, however, lays stress on the effect of tumor stage during restaging TUR with respect to eventual outcome in terms of recurrence and progression rates. A T1 residual tumor on restaging TUR portends failure of conservative therapy with very high recurrence and stage progression rates as compared to patients who have no residual tumor or a Ta stage residual tumor. Moreover, progression in such patients occurs rapidly with the majority showing muscle invasion by 18 months. Since the patients who progress to muscle invasion while on surveillance have a poorer prognosis after radical cystectomy than T1 tumors, operating on such patients before the development of muscle invasion would provide survival benefit.
